# Economics of stem cell transplantation for lymphoma: counting the cost of living

**DOI:** 10.1054/bjoc.1999.0867

**Published:** 1999-12-08

**Authors:** J W Sweetenham

**Affiliations:** CRC Wessex Medical Oncology Unit, University of Southampton, Southampton General Hospital, Tremona Road, Southampton, SO16 6YD, UK


*British Journal of Cancer* (2000) **82**(1), 4–6
© 2000 Cancer Research Campaign
Article no. bjoc.1999.0867

Editorial

### Economics of stem cell transplantation for lymphoma: counting the cost of living

**JW Sweetenham**

CRC Wessex Medical Oncology Unit, University of Southampton, Southampton General Hospital, Tremona Road, Southampton SO16 6YD, UK

High dose therapy (HDT) and autologous stem cell transplantation (ASCT) are now generally accepted as standard salvage therapies for certain patients with relapsed Hodgkin's disease (HD) and aggressive non-Hodgkin's lymphoma (NHL). Evidence for the superiority of HDT and ASCT compared with conventional dose salvage therapy in this setting is based mainly on the results of three small randomized clinical trials together with data from large, retrospective single institution and registry-based studies (Linch et al, 1993; Philip et al, 1995; Schmitz et al, 1999). Although the quality of evidence in favour of ASCT has been questioned (Johnson et al, 1998), it is now such a well-established salvage therapy, that further randomized trials in relapsed patients are very unlikely.

The number of ASCT procedures for lymphoma has increased rapidly over the last decade. Factors contributing to this include the reduced regimen related morbidity and mortality of the procedure with improvements in supportive care, particularly the use of peripheral blood progenitor cells (PBPCs) in place of autologous bone marrow. These improvements have expanded the patient population to whom HDT is applicable, by increasing the upper age limit, and allowing the use of high dose strategies earlier in the course of these diseases. The value of high dose strategies as a component of first-line therapy in these diseases is unclear. Completed randomized trials in aggressive NHL comparing conventional dose therapy with HDT and ASCT in patients who have responded to initial induction chemotherapy have shown no survival advantage for HDT (Haioun et al, 1994; Martelli et al, 1996). However, subset analyses have suggested that certain 'poor risk' patients may benefit from ASCT in first remission (Haioun et al, 1997), and several on-going randomized trials are investigating this issue. If these trials show an advantage for HDT, the potential patient population eligible for this approach will increase substantially. These factors, along with the sustained increase in the incidence of NHL suggest that HDT and ASCT for lymphoma will continue to be a significant burden on health care resources.

Assessment of the impact of this therapy on health care resources is therefore an important, and to date, under-investigated area. In the current issue of the *British Journal of Cancer*, Beard et al (1999) report the results of a study of the cost-effectiveness of HDT and ASCT in relapsed lymphoma, and conclude, in their summary that this is 'both life-saving and cost-effective'. Cost data were derived from figures for drug acquisition and time in hospital at their own institution and applied to the results of published randomized trials. Effectiveness data were derived from these trials, supplemented by survival figures from selected case series of high dose and conventional dose salvage therapy. Using the initial trial data from these trials, they estimate a cost per life year gained (LYG) of £17 375 for relapsed HD, and £12 636 for NHL, with much lower projected costs when the trial data are extrapolated into long-term (10- and 20-year) survival. The authors comment that these figures fall within the accepted UK threshold for treatment that can be regarded as 'cost effective'.

Very few previous studies have investigated economic aspects of HDT and ASCT in comparison with conventional chemotherapy for lymphoma. Uyl-de Groot et al (1995*a*) have reported results from a case-matched study comparing 25 patients with advanced aggressive NHL treated with HDT and autologous bone marrow transplantation (ABMT) during 1992, with a case-matched group receiving conventional dose combination chemotherapy over the same period. Patients were followed for a maximum of 2 years and cost data collected included those for inpatient days, outpatient visits, investigations, drugs, blood product support and hospital overheads. The authors concluded that the use of ABMT had an incremental cost of $27 410 to $37 100 per patient compared with conventional dose therapy. No comparison of effectiveness was made.

The same group undertook a subsequent cost-effectiveness study in patients with aggressive NHL entered onto a randomized trial comparing conventional dose chemotherapy with HDT and ABMT for patients who were 'slow responders' to initial chemotherapy (Uyl-de Groot et al, 1995*b*). Thirty-four patients receiving ABMT were compared with 35 receiving conventional dose therapy. Cost data were collected up to 2 years after completion of therapy. A quality of life study was also conducted in some of the patients, and cost data were collected as in the previous study. Effectiveness was determined using the survival data from the study, extrapolated using a Markov mode. Both life years gained (LYG) and quality-adjusted life years (QaLYs) were also end points. This trial showed no difference in survival for the ABMT arm compared with conventional dose therapy (4-year actuarial overall survival = 85% in the standard arm, and 56% in the high dose arm, $P \geq 0.1$). Overall, the survival and quality of life outcomes were equivalent in both arms, with cumulative hospital costs of $56 512 for ABMT, compared with $20 397 for conventional dose therapy. Based on these results, the authors concluded that ABMT was not cost effective in comparison with standard therapy in this clinical context.

By contrast, the present study, conducted in relapsed disease, suggests a substantial cost-effectiveness advantage to ASCT. Whilst the results of this study are provocative, they should be interpreted cautiously. The clinical trials on which this study is based were small, and only one showed a survival advantage for the high dose arm. The results from single-centre studies incorporated into the effectiveness analysis are subject to potential selection bias, and the populations in these studies may not be comparable. The authors restricted the costs of therapy to predetermined sums for drug acquisition and time in hospital, and have not attempted to calculate costs beyond the date of hospital discharge in patients receiving HDT. Therefore potentially high costs of outpatient therapy and readmission have not been included. All of these are cause for concern in the interpretation of this study.

Estimation of the cost of HDT and ASCT is complex. In addition to the direct costs of drugs and hospital care, there are non-medical direct costs for the patient, including, for example, the cost of transport to and from hospital. Indirect costs such as loss of income of the patient or caregiver are an important factor, as are costs from the societal perspective such as absence from employment. The direct, inpatient costs of HDT and ASCT have fallen in recent years due to improvements in supportive care, and due to advances in the technology of transplantation. Several studies have shown that the use of PBPCs in place of ABMT has resulted in cost savings (Hartmann et al, 1997; Smith et al, 1997). This is mainly due to early haemopoietic recovery, resulting in reduced antimicrobial and blood product support, and earlier hospital discharge. This has been improved further by the use of haemopoietic growth factors following stem cell reinfusion, resulting in even earlier engraftment, with greater economic benefit (McQuaker et al, 1997). The use of high numbers of CD34+ cells is also associated with lower resource use in the inpatient phase of treatment (Schulman et al, 1999). The increasing trend towards out-patient based ASCT is also likely to reduce direct medical costs (Westermann et al, 1999). However, although early hospital discharge and outpatient treatment reduce hospital costs, a proportion of these costs are likely to be transferred to the patient or care-givers. It is essential therefore to consider the cost of increased care-giver burden in determining the overall economic impact of these improvements in therapy.

The experience of an individual transplant centre may also influence the cost of ASCT. In a study from the University of Nebraska, Bennett et al (1995) reported a 'learning curve' phenomenon, whereby the mortality and costs of HDT and ASCT for lymphoma fell over a 5-year period. For example, for patients with Hodgkin's disease, mean costs fell from $96 000 in 1987 to $55 000 in 1991, mainly related to reduced length of hospital stay.

The timing of HDT and ASCT is also likely to be a major factor influencing its effectiveness, and thereby its cost effectiveness. In a decision tree-based analysis of patients with relapsed Hodgkin's disease, Desch et al (1992) have estimated the cost per LYG of HDT and ABMT at $26 000 when used in second relapse, compared with $400 000 when used at first relapse.

These factors underline the complexity of economic analysis of this strategy, and suggest that the type of analysis reported by Beard et al (1999) although interesting, fails to tell the whole story of its cost effectiveness. Perhaps the most important lesson to be drawn from this study is that the value of any economic study is dependent upon the quality of the effectiveness data upon which it is based. In the case of relapsed HD and NHL, few data are available from patients entered into randomized trials.

Further prospective economic studies, preferably in the context of randomized clinical trials are clearly required, and should be conducted according to well described guidelines for cost analysis (NCI Monograph, 1998). It is unlikely that further studies will be conducted in relapsed HD or NHL, where HDT and ASCT are now standard therapy. However, as new studies of ASCT in first remission are developed, where the survival benefits from high dose approaches may be relatively small, cost-effectiveness and cost–benefit studies will take on increasing importance. Inclusion of economic endpoints in these studies is essential. It is also essential that the methods of data collection, analysis and presentation comply with previously published guidelines, to allow consistent interpretation of these data. There is little doubt that the collection of economic data within clinical trials is costly and time-consuming. The increasing 'pressure' on trials groups to include economic analyses in clinical studies must be met by increased resources from funding bodies.

The study by Beard et al (1999) highlights many of the problems of the evaluation of both the effectiveness and the cost of stem cell transplantation. The main priority in this field is to produce high quality evidence for the effectiveness of this treatment modality. While it is too late to revisit this issue for patients with relapsed disease, it is essential that HDT's role as first-line therapy is properly evaluated in prospective trials. Clinical trials to confirm Beard et al's conclusion that HDT and ASCT is 'life-saving' are vital. Calculating the 'cost of living' should be a secondary end point in all of these trials.

### REFERENCES

Beard SM, Lorigan P and Sampson FC (1999) The cost effectiveness of high dose chemotherapy in the treatment of relapsed Hodgkin's disease and non-Hodgkin's lymphoma. *Br J Cancer* **0**: 000–000

Bennett CL, Armitage JL, Armitage GO et al (1995) Costs of care and outcomes for high-dose therapy and autologous transplantation for lymphoid malignancies: results from the University of Nebraska 1987 through 1991. *J Clin Oncol* **13**: 969–973

Desch CE, Lasala MR, Smith TJ, et al. (1992) The optimal timing of autologous bone marrow transplantation in Hodgkin's disease following a chemotherapy relapse. *J Clin Oncol* **10**: 200–209

Haioun C, Lepage E, Gisselbrecht C, et al (1994) Comparison of autologous bone marrow transplantation with sequential chemotherapy for intermediate- and high-grade non-Hodgkin's lymphoma in first complete remission: a study of 464 patients. *J Clin Oncol* **12**: 2543–2551

Haioun C, Lepage E, Gisselbrecht C, et al (1997) Benefit of autologous bone marrow transplantation over sequential chemotherapy in poor-risk aggressive non-Hodgkin's lymphoma: updated results of the prospective study LNH87–2. *J Clin Oncol* **15**: 1131–1137

Hartmann O, Le Corroller A, Blaise D, et al. (1997) Peripheral blood stem cell and bone marrow transplantation for solid tumors and lymphomas: hematologic recovery and costs. *Ann Intern Med* **126**: 600–607

Johnson PWM, Simnett SJ, Sweetenham JW, et al (1998) Bone marrow and peripheral blood stem cell transplantation for malignancy. *Health Technology Assessment* **2**: 8

Linch DC, Winfield D, Goldstone AH, et al. (1993) Dose intensification with autologous bone-marrow transplantation in relapsed and resistant Hodgkin's disease: results of a BNLI randomised trial. *Lancet* **341**: 1051–1054

McQuaker IG, Hunter AE, Pacey S et al. (1997) Low-dose filgrastim significantly enhances neutrophil recovery following autologous peripheral-blood stem-cell transplantation in patients with lymphoproliferative disorders: evidence for clinical and economic benefit. *J Clin Oncol* **15**: 451–457

Martelli M, Vignetti M, Zinzani PL, et al. (1996) High-dose chemotherapy followed by autologous bone marrow transplantation versus dexamethasone, cisplatin and cytarabine in aggressive non-Hodgkin's lymphoma with partial response to front-line chemotherapy: a prospective randomized Italian multicenter study. *J Clin Oncol* **14**: 534–542

NCI Monograph (1998) Integrating economic analysis into clinical trials: the National Cancer Institute–American Society of Clinical Oncology Economics Workbook. Number 24

Philip T, Guglielmi C, Hagenbeek A et al (1995) Autologous bone marrow transplantation as compared with salvage chemotherapy in relapses of chemotherapy-sensitive non-Hodgkin's lymphoma. *N Engl J Med* **333**: 1540–1545

Schmitz N, Sextro M. Pfistner B et al (1999) High-dose therapy (HDT) followed by hematopoietic stem cell transplantation (HSCT) for relapsed chemosensitive

*© 2000 Cancer Research Campaign* 

Hodgkin's disease (HD): final results of a randomized GHSG and EBMT trial (HD-R1). *Proc Am Soc Clin Oncol* **18**: 2a
Schulman KA, Birch R, Zhen B, et al (1999) Effect of $CD34^+$ cell dose on resource utilization in patients after high-dose chemotherapy with peripheral-blood stem-cell support. *J Clin Oncol* **17**: 1227–1233
Smith TJ, Hillner BE, Schmitz N, et al (1997) Economic analysis of a randomized clinical trial to compare filgrastim-mobilized peripheral-blood progenitor-cell transplantation and autologous bone marrow transplantation in patients with Hodgkin's and non-Hodgkin's lymphoma. *J Clin Oncol* **15**: 5–10
Uyl-de Groot CA, Okhuijsen SY, Hagenbeek A, et al (1995*a*) Costs of introducing autologous BMT in the treatment of lymphoma and acute leukaemia in the Netherlands. *Bone Marrow Transpl* **15**: 605–610
Uyl-de Groot CA, Hagenbeek A, Verdonck LF, et al. (1995*b*) Cost effectiveness of ABMT in comparison with CHOP chemotherapy in patients with intermediate and high grade malignant non-Hodgkin's lymphoma. *Bone Marrow Transpl* **16**: 463–470
Westermann AM, Holtkamp MMJ, Linthorst GAM, et al. (1999) At home management of aplastic phase following high-dose chemotherapy with stem-cell rescue for hematological and non-hematological malignancies. *Ann Oncol* **10**: 511–517

*© 2000 Cancer Research Campaign*

